# The Role and Mechanisms of Probiotic Supplementation on Depressive Symptoms: A Narrative Review

**DOI:** 10.1007/s13668-025-00644-1

**Published:** 2025-03-28

**Authors:** Pauline Dacaya, Katerina Sarapis, George Moschonis

**Affiliations:** Discipline of Food, Nutrition and Dietetics, Department of Sport, Exercise and Nutrition Sciences, School of Allied Health, Human Services and Sport, La Trobe University, Melbourne, VIC 3086 Australia

**Keywords:** Probiotics, Microbiota-gut-brain axis, Gut microbiota, Depression, Anxiety

## Abstract

**Purpose of Review:**

The microbiota-gut-brain-axis (MGBA) plays a role in the aetiology of mental disorders. Depression, a leading cause of disability worldwide, may be improved by probiotics. The aim of this narrative review is to investigate and synthesize the current evidence linking probiotic food supplementation with depressive symptomology.

**Recent Findings:**

The gut and the brain communicate and interact via the MGBA through inflammation and the immune system, short chain fatty acid production, neuronal innervation and activation as well as endocrine and neurotransmitter modulation. Dysregulation of gut-brain pathways are caused by gut dysbiosis and implicated in the onset, persistence and exacerbation of depression related symptoms. Modulation of the gut microbiota via administration of probiotics has shown to reduce depressive symptom severity with Bifidobacterium and Lactobacillus strains being the most reported. Probiotics may produce greater benefits in mild depression rather than in chronic, treatment resistant depression.

**Summary:**

Probiotic supplementation is a promising and safe approach for the prevention of severe depressive disorders in high-risk individuals such as people with subthreshold depression. However, the mechanistic pathways of the MGBA require further investigation and additional human clinical trials are necessary to evaluate the role of probiotics on depression.

## Introduction

A mental disorder is characterised by the World Health Organisation [1] as “a clinically significant disturbance in an individual’s cognition, emotional regulation, or behaviour” with the most common being anxiety and depressive disorders. Globally in 2019, approximately 243 million adults experienced anxiety and 257 million adults were living with depression [1]. According to the Australian Institute of Health and Welfare [2], 8.5 million or 43% of Australian adults aged between 16 and 85 have experienced a mental disorder in their lifetime. ‘Mental and substance use disorders’ were the second leading broad disease group, accounting for 15% of the total burden of disease in 2023 [3]. The Australia Bureau of Statistics [4] found mental disorders were a causative factor for approximately 63% of Australian deaths by suicide in 2021 and 4.9% of adults experienced a depressive episode in 2022 [5]. Depressive disorders feature the presence of emptiness, irritability, or sadness in mood which considerably impairs an individual’s ability to function, with the most serious condition in this group of disorders being major depressive disorder (MDD) [6]. Individuals who do not fulfil the diagnostic criteria for MDD but who experience the clinically relevant symptoms may be diagnosed with subthreshold depression [7].

Non-modifiable risk factors commonly linked to depression include gender and genetics. Prevalence rates of anxiety and MDD are almost double in females compared to males [8, 9]. This may be explained by fluctuations in the concentrations of sex hormones estrogen and progesterone throughout the menstrual cycle, pregnancy, post-birth, and transition to menopause [8]. Conversely, adult males with severe depression or dysthymia were found to have lower testosterone levels compared to their healthy counterparts [8]. Subsequently, inheritance of MDD is highly polygenic and involves interaction between multiple loci and environmental triggers [10]. Approximately 180 genetic risk loci and 220 independent single-nucleotide polymorphisms (SNPs) were identified and linked to MDD by the most extensive genome-wide association study on depression [11]. MDD heritability based on SNPs was found to be 11.3% with the biological processes underlying this genetic predisposition involving the development of the nervous system, brain volume, as well as formation and function of the neuronal synapses [11]. In particular, polymorphisms in a serotonin receptor [12] and serotonin transporter gene [13] were found to be linked to subthreshold depression.

Modifiable environmental, lifestyle factors can also pose an important risk or have a protective role in the development of depression. These factors include physical activity, sleep, smoking, exposure to nature, screen time and diet [14]. A Western diet featuring a high intake of saturated fats and sugars has been shown to increase the production of endotoxins and to worsen blood–brain barrier (BBB) leakage and neuroinflammation, thus resulting in decreased cognitive function and depression [15]. In contrast, dietary patterns such as the Mediterranean, Norwegian and Japanese, which are characterized by high intake of whole grains, fruits, vegetables, fish, unsaturated fatty acids, low-fat dairy, olive oil, and antioxidants, have been linked to a lower risk of depression through the reduction of inflammation and increase in beneficial gut microbial taxa [16–18].

Evidence accumulated over the last decade suggests the microbiota-gut-brain axis (MGBA) contributes to the pathophysiology of depression. The MGBA involves the bidirectional communication and interactions between autonomic, central, and enteric nervous systems, circulatory, endocrine, and immune systems as well as the gut microbiota [19, 20]. The gut microbiota refers to the microbe population living within the gastrointestinal tract [21]. During gut dysbiosis, which is characterised by a disturbance to microbiota homeostasis, there is dysregulation between gut-brain pathways [22]. MGBA dysregulation has been implicated in the aetiology of various metabolic and psychological disorders, including depression [23].

Considering the growth in the field of MGBA research, there has been growing interest in gut microbiota modulation via supplementation of probiotics and its effects on mental health. Probiotics are live microorganisms that produce beneficial health benefits via the improvement of host gut microbial balance when administered [22, 24]. Two recent systematic reviews concluded that probiotic supplementation improved depressive symptoms in clinical trials when compared to placebo or control groups [22, 25]. Alli et al. [22] reported that probiotic supplementation treatment may be more beneficial for patients with mild depression rather than patients with chronic, treatment resistant depression. This highlights the preventative role of probiotic supplementation in the manifestation of MDD rather than their role as treatment for the disorder. Another systematic review by Liu et al. [24] found a small but significant effect of probiotics on depression and a significant difference (*p* < 0.01) emerged when analyses were restricted to trials that involved *Lactobacillus* combined with other genera. This is in line with other relevant literature, indicating that the bacterial strains most commonly reported to have anxiolytic effects include *Lactobacillus* and *Bifidobacterium* [26].

MGBA communication occurs through inflammation and immune system activation, the production of short chain fatty acids (SCFAs), endocrine modulation, neuronal innervation and activation as well as the modulation of neurotransmitters. Despite the rapid growth in the research field of the MGBA, the underlying biological mechanisms of the pathways linking probiotic supplementation to depressive symptomatology remain elusive. Therefore, the aim of this narrative review is to synthesize the available evidence related to the mechanisms that link gut microbiota modulation with the pathophysiology of depression.

Relevant literature was identified during April 2024 by searching academic databases including Cinahl, COCHRANE, Web of Science and Medline. The search terms and MeSH terms used were “depression” OR “depressive” OR “depressive symptoms” OR “people with depression” AND “brain-gut axis” OR “gastrointestinal microbiome” OR “gut-brain axis” OR “lactobacillus” OR “microbio*” OR “microbiota” OR “neurotransmitter*” OR “probiotic*” OR “probiotics” OR “receptors, neurotransmitters” AND “anxiety” OR “mental health” OR “mood” OR “psychosocial health” OR “quality of life” OR “stress” OR “stress, psychological.” Peer-reviewed journal articles focused on adults that were published in English since 2014 were included. Grey literature was identified through a manual search of relevant studies and internet search engines.

This review is a narrative synthesis of the association between changes in gut microbiota composition induced by probiotic supplementation and depressive symptoms. The main biological mechanistic pathways were summarised to gain a better understanding of the advancements made over the last decade and to shed light on the potential mediating role of probiotics in the MGBA and mental health.

## Mechanistic Pathways Linking Probiotic Supplementation, Gut Microbiota Modulation and Depression (Fig. 1)

**Fig. 1:**
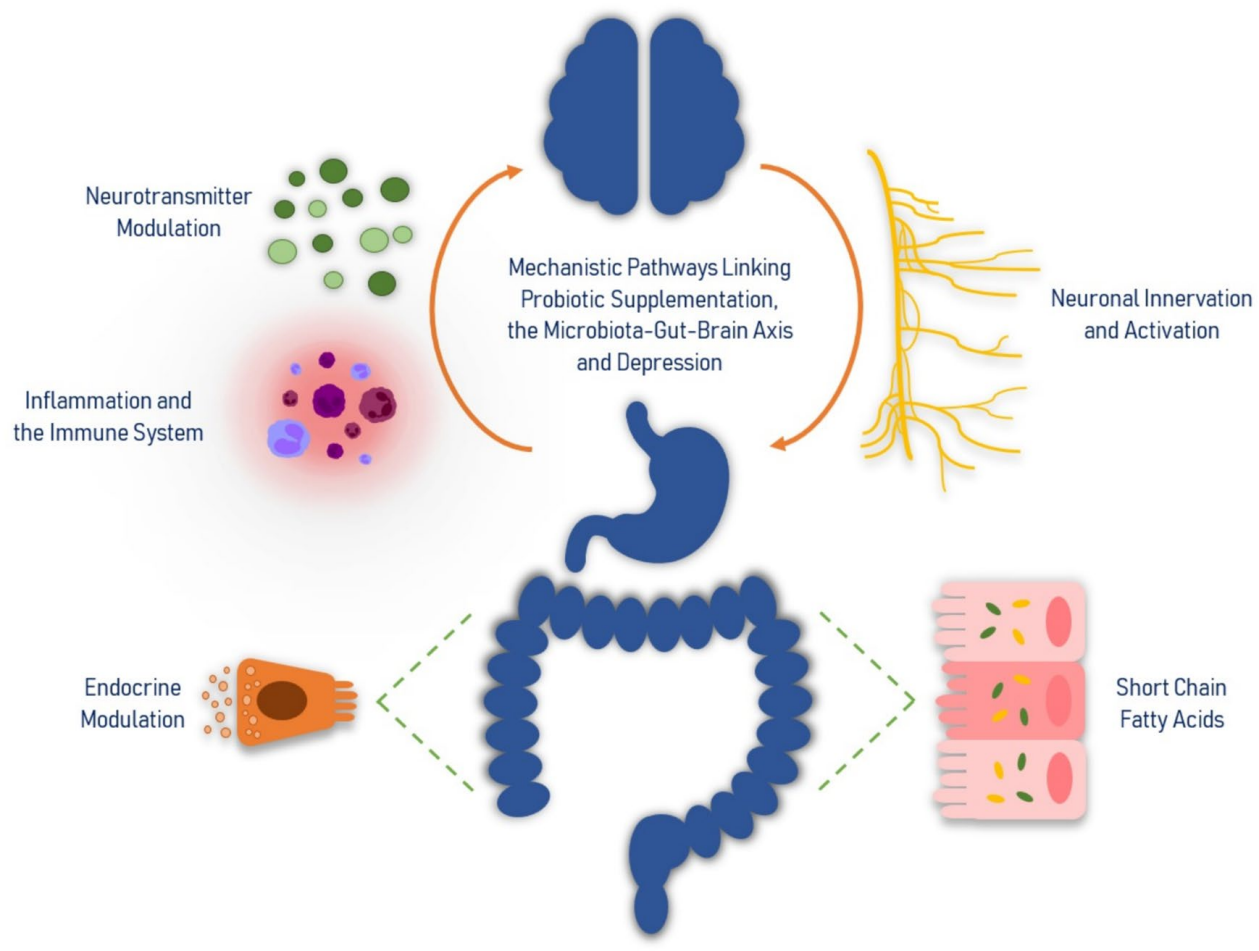
Mechanistic pathways linking probiotic supplementation, the microbiota-gut-brain axis and depression

### Inflammation and the Immune System

MDD is associated with chronic inflammation [23] and activation of the immune system [27]. Evidence suggests that gut inflammation can affect the brain and initiate central inflammation or neuroinflammation via multiple pathways [28, 29]. Alterations in the gut microbiota generate microbial lipopolysaccharide production, triggering inflammatory responses including the production of proinflammatory cytokines, which then activates the afferent loop of the vagal nerve [10, 23]. This stimulates the hypothalamic–pituitary–adrenal (HPA) axis, a key structure in the regulation of the stress response, where chronic stress as well as HPA axis overactivity and dysfunction are proposed to play key roles in MDD development [30].

Peripheral inflammatory molecules are not able to cross the BBB under normal conditions but BBB permeability increases during chronic stress and inflammation [31]. This leads to the excessive translocation of toxic microbial metabolites and immune cells into the brain which enhance brain parenchyma chemokines, cytokines, and endocrine messengers [26]. Depressed patients have been found to have elevated serum levels of proinflammatory cytokine interleukins (IL) (i.e., IL-1, IL-1β, IL-2, IL-6, IL-12), and tumour necrosis factor alpha (TNF-α) as well as reduced levels of anti-inflammatory cytokines (i.e., IL-4, IL-10), and transforming growth factor (TGF)-β1 compared to non-depressed patients [32]. Particularly, increased hippocampal levels of IL-1β have been seen in mice susceptible to stress exhibiting depressive-like behaviours [33]. Proinflammatory cytokines also damage and redirect the activity of tetrahydrobiopterin, an enzyme co-factor necessary for the production of monoamine neurotransmitters including dopamine, norepinephrine, and serotonin as well as activate enzyme indoleamine 2, 3-dioxygenase (IDO), increasing metabolism of serotonin precursor tryptophan via the kynurenine pathway [34]. In depressed subjects, probiotics have shown to regulate cytokine expression and reduce proinflammatory cytokines in mice [35–37] as well as upregulate genes associated with immune activation and decrease inflammatory biomarkers in humans [38, 39].

In addition to alterations of inflammatory signals and proinflammatory cytokines in people with gut dysbiosis, immune cells are also activated and migrate into the brain [34]. Among these cells, microglia are the immune cells residing within the central nervous system (CNS) whose development and activity is altered in response to gut microbiota composition changes [28, 29]. Microglial activation contributes to neural toxicity via the release of reactive nitrogen and oxygen species, chemokines, and cytokines into extra-synaptic space leading to a compromised BBB, dysregulation of neurotransmitter systems, imbalance of the excitatory to inhibitory ratio, damage to epithelial cells of the brain as well as disruption to the plasticity and adaptation of neural circuitry [40, 41].

Neuroinflammation impacts up to 27% of MDD patients and is linked to poor prognosis, resistance to treatment, and reduced quality of life [31]. Long-term neuroinflammation disrupts brain function and may influence mood and behaviour [10], thus increasing the risk for depression. Depression exacerbates cytokine responses and this results in a vicious cycle where inflammation and depression fuel one another [10].

### Short Chain Fatty Acids

SCFAs are organic carboxylic acids with up to six carbon atoms [25] and are neuroactive metabolites of the microbiota that facilitate communication between the gut and the brain [42]. SCFAs reach systemic circulation and pass through the BBB via specialised transporters found on brain vascular epithelial cells to affect crosstalk between neurons and immune cells that play a role in behaviour and brain function [15, 43]. Under physiological conditions, low amounts of SCFAs are detectable in the human brain [44].

SCFAs may exert a protective effect against depression via multiple mechanisms. First, SCFAs play a role in immune activity through the production of chemokines and cytokines and during the immune response, stimulation of T cell differentiation into anti-inflammatory T regulatory (Treg) cells [45], regulation of microglial maturity and function, and can also act as histone deacetylase inhibitors which have been considered as potential novel antidepressants due to their anti-inflammatory and immunosuppressive properties [46]. Second, SCFAs maintain gut homeostasis by strengthening intestinal barrier integrity of colonic cells and promoting mucus production which decreases permeability, preventing microbial endotoxins from entering circulation, thereby preventing initiation of an immune response and uncontrolled inflammation [46, 47]. Third, SCFAs can activate G protein-coupled receptors involved in the production of neurotransmitters [48] and promote the synthesis of serotonin in enterochromaffin cells [49], while they can also modulate neurotransmitters including gamma-aminobutyric acid (GABA), glutamate, glutamine, and neurotrophic factors [50].

SCFAs, such as butyrate and propionate, are produced by microbes in the gut and can regulate tryptophan 5-hydroxylase 1 expression involved in serotonin production, and tyrosine hydroxylase involved in the synthesis of adrenaline, noradrenaline, and dopamine [51]. Compared to healthy controls, depressed patients have shown a decrease in anti-inflammatory butyrate and an increase in proinflammatory lipopolysaccharides (LPS) [15]. Probiotic administration has raised acetate and butyrate levels whilst reducing anxiety and stress in rodents [37]. Moreover, butyrate alters the expression of brain-derived neurotrophic factor (BDNF), which has a crucial role in brain cell survival as well as synaptic structure and plasticity [52]. The mutation of BNDF or inhibition of the cAMP signalling pathway affecting BDNF in mice seem to predispose the development of depression-like behaviour whereas the stimulation of the BDNF signalling pathway in the hippocampus has resulted in antidepressant-like activity [53]. A number of preclinical studies have resulted in increased levels of BDNF and decreased anxiety or depressive symptoms following probiotic administration [37, 54–57]

### Neuronal Innervation and Activation

A prominent component of the MGBA is the vagus nerve which links the gut microbiota to the CNS [34]. The vagus nerve is involved in the regulation of hormone release, immune function and inflammatory responses, metabolic homeostasis, and neural transmission [15, 34]. Microbial metabolites have shown to activate the vagus nerve by stimulating sensory neurons of the enteric nervous system [58, 59]. The vagus nerve senses proinflammatory cytokines and relays this information to the brain where the HPA axis is activated to suppress the immune response [34] which includes inhibiting the release of proinflammatory cytokines from the gut [15].

Preclinical studies performed on mice have shown the probiotic species of *Bifidobacterium longum* and *Lactobacillus rhamnosus* are able to exert their beneficial effects on depression and anxiety if the vagus nerve is intact and not in vagotomized mice [60, 61]. This highlights that the vagus nerve is integral for communicating information regarding the bacterial contents of the gut to the brain [62]. The vagus nerve partially mediates the anxiety and depression reducing effects of probiotic strains via the modulation of neural transmission and may also influence neuroendocrine activity [34].

### Endocrine Modulation

The activity of enteroendocrine cells (EECs) are also modulated by the gut microbiota [63]. Bacterial composition and diversity of the enteric system affect the release of gut peptides such as cholecystokinin (CCK), corticotropin-releasing factor (CRF), ghrelin, glucagon-like peptide (GLP-1), peptide YY (PYY), and oxytocin [64]. Not only do most gut-derived peptides participate in the regulation of appetite and intake of food but the roles of peptides in the brain are also well-established in the neurobiology of anxiety and depression [65]. Alterations in the gut microbiota are likely to modulate the expression of gut-derived peptides and peptide hormones, which may have important roles in the communication between the gut and the brain [66–68]. Probiotics have been administered as a means to alter gut microbiota composition in mice and resulted in decreased anxiety, depression, plasma and intestinal GLP-1 [69], which in addition to its role in the regulation of body weight and food intake, GLP-1 is also involved in the overall stress response [64].

According to human clinical trials, depressed patients have shown that HPA axis activation can alter gut microbiota composition and induce inflammation [10]. Specifically, the HPA axis secretes cortisol during the stress response which promotes intestinal barrier permeability to Gram-negative bacteria [70]. Probiotic administration in stressed mouse models has been shown to reduce corticosterone levels [71, 72] and restore gut barrier integrity [37, 47]. Immune responses are triggered by the release of bacterial toxins and waste products into systemic circulation resulting in the HPA axis upregulating cortisol production [46]. This emphasises the vicious cycle of increased stress levels induced by depression, leading to unfavourable changes in gut microbiota composition, which go on to negatively affect mood.

### Neurotransmitter Modulation

Gut microflora can affect brain function and mood via the production and modulation of neurotransmitters [73, 74]. The gut microbiota can also synthesize and regulate the absorption and function of soluble factors such as neuromodulators [62]. Cell culture studies have shown that gut microbiota can produce neurotransmitter precursors such as tryptamine, and also directly synthesise neurotransmitters including dopamine, GABA, norepinephrine, and serotonin [75–77]. Specifically, bacteria from genera *Bifidobacteria*, *Enterococcus, Lactobacillus,* and *Streptococcus* have shown to produce acetylcholine, GABA, and serotonin which directly and indirectly influence the physiology of brain cells [74, 78, 79].

Both GABA and serotonin play a role in regulating mood, since low levels of these inhibitory neurotransmitters are linked with anxiety and depression [52]. Oral administration of *Lactobacillus rhamnosus* has shown to modulate the expression of GABA receptors in the amygdala, prefrontal cortex and hippocampus to reduce behaviours related to anxiety and depression in mice [61]. In human trials, GABA has been efficiently produced by *Bifidobacterium* which is found to be decreased in the gut microbiota of MDD patients compared to controls [48].

Regarding other key neurotransmitters, over 95% of serotonin is produced in the gut and is associated with various psychological states including sleep, behaviour, and mood [78, 80]. Serotonergic fibers densely innervate the HPA axis which plays an integral part in stress response regulation [8] but it should be noted that plasma serotonin is unable to pass through the BBB [20, 80]. Preclinical trials on chronic, mild depression have shown reduced concentration, expression, neurotransmission and release of serotonin in the medial prefrontal cortex and hippocampus [81]. Likewise, significant decreases in serotonin and specific SCFAs are associated with gut microbiota dysbiosis in depression [15]. Recent novel trials in humans using multi-strain probiotic supplementation has shown increased levels of serotonin at 6 weeks along with improvements in mood, anxiety, and depression [80], followed by decreased serotonin from 6 to 12 weeks [82]. This suggests that changes to the gut microbiota may modulate serotonin signalling pathways in the brain that can subsequently affect mental health.

All studies included in this narrative review are summarized in Table 1.

**Table 1:** Summary of included studies

Author(s)	Year	Country	Type of Study	No. of Subjects	Summary of Clinical Findings
Abildgaard et al. [36]	2017	Denmark	Animal	40 rats	Probiotic treatment reduced depressive-like behaviour independently of diet
Akkasheh et al. [39]	2016	Iran	RCT	40	Probiotics reduced depression severity and inflammatory markers
Alli et al. [22]	2022	Various	Systematic Review	3,936	Probiotics benefit mild depression more than chronic treatment-resistant depression
Anand et al. [26]	2023	Various	Review	N/A	Gut dysbiosis contributes to neuropsychiatric disorders, including depression
Ait‐Belgnaoui et al. [47]	2014	France	Animal	N/A	Probiotic supplementation prevented stress-induced brain activity abnormalities in mice
Aoki et al. [69]	2016	Japan	Animal	N/A	*Lactobacillus casei Shirota* improved anxiety-like and depressive behaviours in mice
Beurel et al. [27]	2020	Various	Meta-analysis	36 mice	Depression linked to inflammation; probiotics may help modulate immune response
Dhaliwal et al. [37]	2018	India	Animal	60 mice	*Lactobacillus plantarum* supplementation improved depression-like behaviour in mice
Gonda et al. [13]	2021	Hungary	Observational	128	Subthreshold depression associated with serotonin transporter gene polymorphism
Han et al. [55]	2019	South Korea	Animal	48 mice	*Lactobacillus mucosae* and *Bifidobacterium longum* alleviated stress-induced anxiety and depression via gut dysbiosis suppression
Han et al. [57]	2020	South Korea	Animal	35 mice	*Lactobaccillus reuteri* and *Bifidobacterium adolescentis* alleviated gut dysbiosis and reduced stress-induced depression
Jiang et al. [70]	2015	China	Observational	46	Depressed patients had altered gut microbiota composition
Liang et al. [54]	2015	China	Animal	32 rats	*Lactobacillus helveticus* had antidepressant and anxiolytic effects in mice via the microbiota-gut-brain axis
Liu et al. [72]	2016	Taiwan	Animal	N/A	*Lactobacillus plantarum* PS128 improved anxiety and depressive-like behaviour in mice
Liu et al. [24]	2019	Various	Meta-analysis	34 trials	When combining *Lactobacillus* with other strains, probiotics significantly improved depression
Maybee et al. [19]	2022	Various	Systematic Review	157 studies	Probiotic supplementation was safe and effective in reducing depressive symptoms
McVey et al. [71]	2018	Canada	Animal	46 mice	*Lactobacillus rhamnosus* reduced corticosterone levels in mice
Mohan et al. [23]	2023	Various	Review	N/A	Altered gut microbiome linked to depression; potential for probiotic intervention
Morais et al. [20]	2021	Various	Review	N/A	The gut microbiota influences neuropsychiatric disorders via complex microbiota-gut-brain axis mechanisms
Moschonis et al. [82]	2024	Australia	RCT	72	Multi-strain probiotics increased serotonin concentrations at 6 weeks, followed by a decline at 12 weeks
Ng et al. [73]	2018	Various	Meta-analysis	1,349	Probiotics significantly improved the mood of individuals with mild to moderate depressive symptoms
Pokusaeva et al. [79]	2017	USA	RCT	129 and 30 mice	*Bifidobacterium dentium* influenced GABA production in humans and modulated brain function
Sanada et al. [25]	2020	Various	Meta-analysis	1,003	Probiotics significantly improved depressive symptoms in MDD patients when compared to controls
Sempach et al. [38]	2024	Switzerland	RCT	43	Multi-strain probiotics modulated immune response and reduced inflammation to improve depression
Tian et al. [56]	2022	China	Animal	30 mice	*Bifidobacterium* strains significantly reduced depressive behaviours in mice via serotonin and gut microbiota regulation mechanisms
Walden et al. [80]	2023	USA	RCT	70	Multi-strain probiotics improved depression and increased serotonin concentrations at 6 weeks
Yoo et al. [35]	2022	South Korea	RCT	10 and 6 mice	Probiotics reduced inflammation and depression in mice via the regulation of cytokine expression
Yunes et al. [76]	2016	Russia	RCT	N/A	More than 40% of human gut-derived *Lactobacillus* and *Bifidobacterium* strains synthesise and produce GABA

*GABA*, gamma amino-butyric acid; *MDD*, major depressive disorder; *RCT*, randomised controlled trial

## Conclusion

Mainly preclinical but also some clinical trials suggest that gut dysbiosis plays a role in the cause, persistence and worsening of neuropsychiatric disorders. The gut and the brain communicate and interact via various complex and interrelated pathways. Gut microbiota alteration via the administration of probiotics may contribute to gut homeostasis and therefore, the reduction of anxiety and depression-related symptoms.

A notable strength identified through this review is that there were no clinically significant adverse events reported after probiotic administration by systematic reviews conducted by Maybee et al. [19] and Ng et al. [73] involving a combined total of 167 studies. Additionally, probiotics were found to be more effective in reducing depressive symptoms in MDD patients compared to prebiotics and postbiotics [23].

Conversely, studies were limited by small sample sizes, a lack of consideration for diet or antidepressant treatment effects, differing microbiome sequencing methodology and regional variations, wide variations in gut microbiome composition due to age [27] and race (as trials were conducted in a variety of nations), a lack of consistency in specific bacterial strains and colony-forming unit CFU counts [19], and differing modes of delivery including conventional methods (i.e. capsules, powders and tablets) and non-conventional methods (i.e. cheese, milk, and yoghurts) [73]. It is important to consider that probiotics must endure gastric transit to promote beneficial effects in the intestine, and that processing techniques affect bacterial survival and viability [73].

Overall, the exploratory nature of this narrative review has allowed for the critical synthesis of recent literature and provided a broad overview of relevant emerging areas of research. Although non-exhaustive, the findings suggest that despite some significant advancements in understanding the molecular mechanisms of depression, the exact mechanistic pathways remain unclear and have been predominantly examined in animal models. Equally, the field of MGBA research is still young. In this regard, further investigation is required in humans to fully understand the mechanistic pathways that link probiotic supplementation and the modulation of the gut microbiota with mental health and mood disorders.

## Data Availability

No datasets were generated or analysed during the current study.
